# Effects of a Playoff Match on Competitive Anxiety and Autonomic Regulation in Professional esports Players

**DOI:** 10.2174/0117450179293069250507074009

**Published:** 2025-05-22

**Authors:** Sergio Machado, Leandro de Oliveira Sant’Ana, Luis Cid, Diogo S. Teixeira, Antonio Egidio Nardi, Bruno Travassos, Diogo Monteiro

**Affiliations:** 1 Laboratory of Panic and Respiration, Institute of Psychiatry (IPUB), Federal University of Rio de Janeiro (UFRJ), Rio de Janeiro, Brazil; 2 Department of Sports Science, University of Beira Interior, 6201-001 Covilhã, Portugal; 3 Laboratory of Physical Activity Neuroscience, Neurodiversity Institute, Queimados 26325-020, Brazil; 4 Postgraduate Program in Physical Education, Federal University of Juiz de Fora, Juiz de Fora, Brazil; 5 Research Center in Sport, Health and Human Development (CIDESD), 5000-558 Vila Real, Portugal; 6 Sport Sciences School of Rio Maior, Polytechnic of Santarém (ESDRM-IP Santarém), Santarém, Portugal; 7 Life Quality Research Centre (CIEQV), 2400-901 Leiria, Portugal; 8 Faculty of Physical Education and Sport, Lusófona University, 1749-024 Lisbon, Portugal; 9 Research Center in Sport, Physical Education, and Exercise and Health (CIDEFES), (CIDEFES), 1749-024 Lisbon, Portugal; 10 ESECS, Polytechnic of Leiria, 2411-901 Leiria, Portugal; 11 Portugal Football School, Portuguese Football Federation, 1495-433 Cruz Quebrada, Portugal

**Keywords:** Victory, Defeat, Esports, Competitive anxiety, Heart rate variability, HRV

## Abstract

**Introduction:**

A competition is considered a stressful situation since it causes physiological and emotional changes in the responses of athletes and consequently influences their performance. The aim of our study was to investigate competitive anxiety and heart rate variability (HRV) in professional eSports athletes, comparing the responses before and after matches based on whether they won or lost. We hypothesized that victorious players would display more favorable autonomic and anxiety-related responses after the matches compared to those who were defeated.

**Methods:**

We recruited fifty male esports players from 10 different Brazilian teams and carried out the experiment across two sessions. Initially, 24 hours before the game, players signed the informed consent form, and sample characterization, along with player familiarization with anxiety and HRV, was performed. Following this, the players recorded their anxiety levels and HRV at rest for 10 minutes, both 60 and 30 minutes prior to the game (baseline time points), as well as 10 minutes after the conclusion of the game.

**Results:**

Regarding anxiety, our results demonstrated that the victory group (VG) exhibited significantly lower scores for both cognitive and somatic anxiety in the post-game time point, coupled with increased scores for self-confidence when compared to the baseline (BL) and pre-game time points. In opposition, the defeated group (DG) displayed significantly elevated scores for cognitive and somatic anxiety during the post-game time point, accompanied by decreased self-confidence scores compared to the baseline and pre-game time points. Regarding heart rate variability (HRV), the victory group (VG) demonstrated a significant increase in SDNN, rMSSD, and HF measures, coupled with a significant decrease in the LF/HF ratio. Conversely, the defeated group (DG) exhibited a significant decrease in SDNN and rMSSD, along with a significant increase in the LF/HF ratio.

**Discussion:**

Our results revealed that VG exhibited better HRV responses, indicating greater parasympathetic activation. VG also showed lower levels of cognitive and somatic anxiety and higher levels of self-confidence in the post-game time. In contrast, DG demonstrated worse HRV responses, indicating greater sympathetic activation, along with higher levels of cognitive and somatic anxiety and lower levels of self-confidence in the same post-game period.

**Conclusion:**

In summary, the VG exhibited superior HRV responses in conjunction with lower levels of anxiety compared to the DG.

## 1 INTRODUCTION

Competition is considered a stressful situation since it causes physiological and emotional changes, such as anxiety, in the responses of the athletes and consequently influences their performance [1]. Anxiety is inherent to sports, as it can be experienced at different levels [2]. A higher state of anxiety that occurs before or during sports competitions is defined as competitive anxiety [3], which can be classified as cognitive and somatic anxiety [4].

Cognitive anxiety is characterized by negative expectations about performance success or self-evaluation, accompanied by adverse feelings, thoughts, fears, and worries regarding one's performance. This can lead to a decline in self-esteem and self-confidence, negative self-talk, difficulty concentrating, and disrupted attention. In contrast, somatic anxiety is characterized by heightened physiological arousal, including increased heart rate and muscle tension. This can result in negative symptoms, such as nervousness, breathing difficulties, elevated blood pressure, and muscle tightness [4]. These changes in emotional states can occur before, during, or after sports competitions [2]. Thus, an increase in anxiety can lead to negative consequences in the athletes’ performance [5, 6]. There is a scarcity of studies on esports competition as a whole, and the impact of a match on physiological and psychological variables in professional esports players is of great interest to researchers and coaches [7, 8]. As the sport continues to evolve rapidly, gaining insights into its psychophysiological aspects will be crucial for informing technical, tactical, and training-related adjustments.

Esports, better known as electronic sports, are competitions involving video games, *e.g.*, League of Legends, and their participants, referred to as players, may compete at amateur or professional levels [8]. From a psychophysiological point of view, the autonomic nervous system (ANS) shows changes in sympathetic and parasympathetic activities [9]. Considering the sports context, the ANS assessment can be performed non-invasively through heart rate variability (HRV) [10]. In fact, HRV is a sensitive physiological indicator for psychological regulation [11] that detects changes in sympathetic/parasympathetic balance in stressful situations, such as sports competitions [12].

Research carried out by Mateo *et al.* [13] and Bernston *et al.* [14] reported that HRV is a sensitive indicator of changes in emotional state, thus suggesting an interaction between cardiac and neural processes. It is well known that cardiac control and response to different stressors can be explained by both variations in activation of the sympathetic nervous system (SNS) [15] and parasympathetic nervous system (PNS) [16-18]. In line with this, there is abundant evidence showing that a decrease in HRV parameters is associated with anxiety [19-21]. For example, Friedman [16] and Cohen and Benjamin [22] revealed that reductions in HRV parameters correspond to an increase in anxiety. These findings prompt us to explore the impacts of match outcomes on competitive anxiety and heart rate variability (HRV). This is important, as esports appear to rely much on psychological and neurocognitive abilities for success compared to traditional sports [8]. From a practical perspective, some studies addressed competitive anxiety [23-26] and HRV responses [27] to successful or unsuccessful match outcomes in traditional sports. However, there are no studies addressing competitive anxiety and HRV responses to successful or unsuccessful match outcomes (i.e., victory and defeat) in professional esports players. A few similar studies were conducted by Pena [27] and Fuentes-Garcia *et al.* [28], both on tennis players. Pena [27] investigated HRV before and after competition in junior tennis players. Results indicated that losers showed a reduction in PNN50 both on the training day and after the match compared with winners. A study by Fuentes-Garcia *et al.* [28] examined the influence of match results (win or loss) in an international competition on pre-competition anxiety and self-assurance among elite junior tennis players. The research revealed substantial differences in cognitive anxiety and self-confidence between victors and defeated athletes when comparing their pre-match and post-match values. More specifically, the post-match evaluations of the victorious athletes revealed a reduction in cognitive anxiety and an increase in self-confidence. Regarding esports, the existing studies mainly deal with reports of playing video games or have analyzed the lifestyles and health-related issues of the players. Thus, there is a need to understand the variations in cardiovascular, respiratory, metabolic [29], and psychological health [30] in esports in different contexts or according to different game results. The existing academic literature lacks any published research that specifically examines the impacts of both victory and defeat on competitive anxiety and heart rate variability (HRV) among professional esports athletes. A better understanding of competitive anxiety and HRV behaviors could contribute to enhancing athletic performance [31-34], thus allowing for the assessment of the recovery process between matches and throughout the competition [35].

The objective of this study was to analyze the impact of the outcome of a playoff game (victory and defeat) on the levels of competitive anxiety and HRV in professional esports athletes. More precisely, the study tested the following hypothesis: victorious players will exhibit lower levels of both cognitive and somatic anxiety, higher self-confidence, and superior autonomic responses compared to defeated athletes in the aftermath of the match.

## 2 METHODS

### 2.1 Participants

The required sample size for this study was calculated using the G*Power v.3.1 software [36]. The input parameters used in the power analysis were a medium anticipated effect size for a comparison between two dependent means (d = 0.50), a statistical power (1-β) set at 0.80, and a significance level (α) of 0.05. Since no previous studies had provided results directly relevant to the targeted effect in our research, the researchers instead used effect size estimates from similar experimental manipulations involving psychophysiological responses in the context of competitive stress within sports science [37]. Notably, the effect sizes observed in the referenced study were found to be large [37].

The target sample size determined for our study was 20 participants (1-β = 1.616), supported by power calculations. Fifty male esports players between the ages of 18 and 29 years (mean age: 24.98 ± 2.59 years, height: 178.6 ± 1.45 cm, weight: 78.5 ± 2.35 kg, experience duration: 7.68 ± 1.33 years, and physically inactive for 36 ± 1.05 minutes per week) from several Brazilian teams took part in the study. To be eligible, participants had to be professional athletes with at least 5 years of experience in national or international competitions and engaged in 8 to 10 hours of daily training. Exclusion criteria included having neuropsychiatric, cardiovascular, or osteoarticular diseases, using neuropsychiatric drugs, and consuming caffeinated or alcoholic beverages on the day of the experiment. The study was approved by the Ethics Committee of the University of Beira Interior (D2574), and all athletes were instructed about the risks and benefits related to the study before providing written informed consent.

### 2.2 Experimental Design

Participants were recruited from 10 distinct Brazilian teams, each comprising 5 players. If any individual failed to satisfy the inclusion criteria, the entire team was omitted from the study. The research methodology entailed conducting the study with two teams per pivotal game, enabling equal observation of the phenomena of triumph and defeat. As a result, upon completing the experiment, there were 25 players in the victory group (VG) and defeated group (DG). Players were exposed to pivotal games in international tournaments, such as the CS: GO PGL Major Championship Fall and Six Invitational. The study unfolded across two sessions.

In the first session, 24 hours before the game, players signed the informed consent form in the game house. Participant characteristics, such as anthropometric data (age, stature, and body mass), physical activity levels evaluated through the International Physical Activity Questionnaire (IPAQ) [38], and expertise time, were assessed for sample description. Furthermore, the Revised Competitive State Anxiety Inventory 2 (CSAI-2R) and heart rate variability (HRV) assessments were carried out in individuals.

**Fig. (1):**
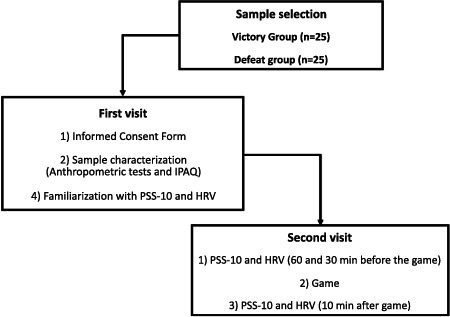
Experimental design.

During the second session, participants completed the CSAI-2R [39] and had their heart rate variability (HRV) recorded at rest for 10 minutes, 60 minutes, and 30 minutes prior to the match (baseline [BL] and pre-game time points, respectively) as well as 10 minutes after the conclusion of the match (post-game time point, Fig. **1**). Both sessions took place at the gaming residence between 14:00 – 17:00 to mitigate the influence of circadian rhythms on psychological and autonomic function. An investigator evaluated the psychological variables and HRV.

### 2.3 Familiarization

The familiarization process with the CSAI-2R was conducted by the same investigator 24 hours before the experimental conditions, and it involved the following steps: 1) the researcher provided a thorough reading of the detailed instructions for each query; 2) It was clarified that responses should be selected from the options offered and that there are no right or wrong answers; 3) players were told not to answer the same question again, stressing the value of being truthful in their comments; 4) Prior to finishing the questionnaire, players were instructed to review their responses.

The familiarization process took place only prior to the experimental phase. The players then individually completed the questionnaire during the experimental conditions. Players were instructed to use the heart rate monitor, position the chest strap over the xiphoid process, stay still, keep their eyes open, and breathe normally during the acquisition period in order to acquire HRV data.

### 2.4 Revised Competitive State Anxiety Inventory 2 (CSAI-2R)

To evaluate pre-competitive anxiety, participants completed the Revised Competitive State Anxiety Inventory 2 (CSAI-2R) questionnaire. This instrument, developed by Cox *et al.* [40], is a condensed version of the CSAI-2 proposed by Martens *et al.* [41]. The CSAI-2R comprises 17 questions categorized into three components: cognitive anxiety (7 items), somatic anxiety (5 items), and self-confidence (5 items). The cumulative score for each sub-scale is determined by summing the Likert points obtained for each item. Each question offers

four response alternatives using a Likert scale ranging from 1 (strongly disagree) to 4 (strongly agree). The Brazilian adaptation of CSAI-2R demonstrated favorable psychometric properties for evaluating pre-competitive anxiety in Brazilian athletes [39]. The assessment duration was limited to 10 minutes. In our sample, the Cronbach's α values were 0.76, 0.81, and 0.79 for the sub-scales of cognitive anxiety, somatic anxiety, and self-confidence, respectively.

### 2.5 Heart Rate Variability

HRV information of all players was recorded and analyzed while they were seated on a bench in a room with air conditioning [42]. Once the assessment of competitive anxiety was completed (approximately 5 minutes), the players were provided with moistened strap transmitters, which were securely fastened to their chests. Subsequently, the players verified that the receiver of the heart rate monitor was functioning properly in order to acquire RR intervals [43]. The HRV measurements were taken at rest using a Polar V800 cardiotachometer (Polar™, Kempele, Finland) with a sampling rate of 1,000 Hz [44]. HRV was recorded for 10 minutes, which included 5 minutes for stabilization and five minutes for post-stabilization. Using specialized software, data pertaining to the 5 minutes following stabilization was collected and downloaded for analysis (Polar Precision Performance, PolarTM, Kempele, Finland). The 5-minute post-stabilization HRV indicators were examined using the KubiosTM HRV program (Biomedical Signal Analysis Group, Department of Applied Physics, University of Kuopio, Kuopio, Finland) [45]. Artifacts (≤ 2%) in the data were visually inspected and manually removed with the use of interpolated adjacent RR interval values (filter power < medium) [46]. The variables were reliant on the time domain (standard deviation of the mean of the qualifying NN interval) and frequency domain (high frequency (HF) and sympatho-vagal balance (LF/HF)).

### 2.6 Statistical Analyses

Shapiro-Wilk and Levene’s tests were carried out to check the homogeneity and normality, respectively. As observed in the normality tests, the data followed a normal distribution. The information was displayed using mean and standard deviation (M ± SD). At the initial stage (BL), the criteria for age, weight, height, time of experience, CSAI-2R, and HRV were satisfied. Consequently, independent samples t-tests were carried out to compare distinctions between the two groups (VG *vs* DG) at the BL. A 2 × 2 mixed-factor analysis of variance (ANOVA) was utilized to determine variances between the VG and DG (between-group effects) and changes across the baseline, pre-game, and post-game periods (within-group effects) for CSAI-2R and HRV measurements in both the time and frequency domains. Subsequent analysis was performed using the Bonferroni method to assess the impacts within each group. The significance level was established at 5% (p < 0.05). Effect sizes were computed and interpreted according to Cohen's d guidelines: 0 .00 to 0 .19 (trivial), 0 .20 to 0 .49 (small), 0 .50 to 0 .79 (moderate), and ≥0.80 (large) [47]. Pearson's correlation analysis (bivariate) was carried out to examine the relationship between CSAI-2R and HRV measures [48]. All statistical analyses were performed using GraphPad Prism software, version 8.0.1.

## 3 RESULTS

The analyzed groups exhibited homogeneity as there were no significant differences between the groups in terms of age (25.04±2.77 *vs*. 24.92±2.41 years old, p = 0.87), weight (79.6 ± 2.1 *vs*. 77.4 ± 2.6 Kg, p = 0.78), height (179.5 ± 1.6 *vs*. 177.7 ± 1.3 cm, p = 0.69), time of experience (7.92 ± 1.29 *vs*. 7.44±1.38 years, p = 0.22), and physical activity level (37 ± 1.2 *vs*. 35 ± 0.9 min per week, p = 0.63).

### 3.1 Revised Competitive State Anxiety Inventory 2 (CSAI-2R)

**Fig. (2):**
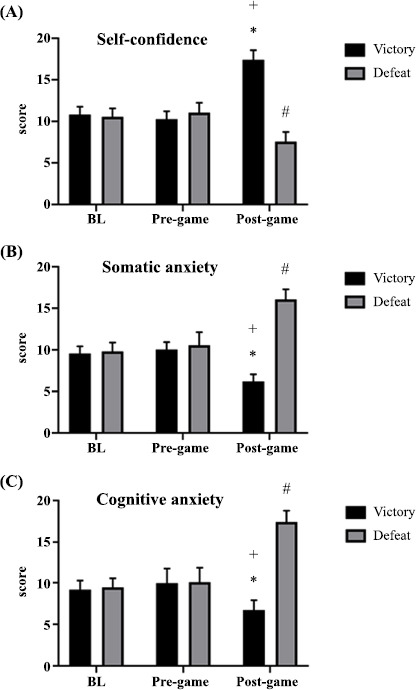
Anxiety patterns in both groups. (**A**) *Significant difference compared to BL and pre-game times (p ≤ 0.0001), **^+^**Significant difference compared to post-game time (p ≤ 0.0001), #*Significant difference compared to BL and pre-game times (p ≤ 0.0001); (**B**) *Significant difference compared to BL and pre-game times (p ≤ 0.0001), **^+^**Significant difference compared to post-game time (p ≤ 0.0001), #*Significant difference compared to BL and pre-game times (p ≤ 0.0001); (**C**) *Significant difference compared to BL and pre-game times (p ≤ 0.0001), **^+^**Significant difference compared to post-game time (p ≤ 0.0001), #*Significant difference compared to BL and pre-game times (p ≤ 0.0001).

There were no significant differences between groups in terms of self-confidence at the baseline (p = 0 .999) and pre-game (p = 0.184) periods. Additionally, there were no within-group differences between the baseline and pre-game times for the VG (p = 0 .754) and the DG (p = 0 .844). Nevertheless, in the post-game period, the Competitive State Anxiety Inventory-2 Revised (CSAI-2R) scores were higher in the VG group compared to the DG group (p ≤ 0.0001; effect size d = 2.26, 95% confidence interval: 1.52 to 2.93, (Fig. **2A**). A mixed analysis of variance revealed a significant interaction between group and time (F(2, 144) = 370.7; p ≤ 0.0001), main effects for the group (F(1, 144) = 313.4; p ≤ 0.0001), and time (F(2, 144) = 47.28; p = 0.003) for anxiety state.

The interaction analysis showed increased levels of self-confidence post-game (17.40 ± 1.15) compared to BL (9.79 ± 1.53) and pre-game (10.78 ± 0.92) in the VG (p ≤ 0.0001; d = 5.62, CI 95%: 4.32 to 6.75 and d = 6.36, CI 95%: 4.91 to 7.60, respectively). On the other hand, there was a decreased self-confidence score post-game (8.1 ± 5.7) compared to BL (10.52 ± 1.12) and pre-game (10.79 ± 1.53) in the DG (p ≤ 0 .0001; d = 0 .59, CI 95%: 0.01 to 1.15 and d = 0.64, CI 95%: 0.07 to 1.20, respectively).

For somatic anxiety, no differences were found between groups at BL (p = 0.827) and pre-game (p = 0.265). Additionally, no intra-group differences were found between BL and pre-game in the VG (p = 0.867) and DG (p = 0.215). CSAI-2R in the post-game time was lower in VG than in DG (p ≤ 0.0001; d = 9.59, CI 95%: 7.51 to 11.37, Fig. **2B**). The mixed analysis of variance showed a significant group-by-time interaction (F(2, 144) = 309.4; p ≤ 0.0001), main effects for group (F(1, 144) = 387.9; p ≤ 0.0001), and time (F(2, 144) = 22.08; p ≤ 0.0001) for anxiety state. The interaction analysis revealed a reduction in somatic anxiety levels post-game (6.16 ± 0 .86) compared to BL (9.58 ± 0 .88) and pre-game (10.04 ± 0 .90) in the VG (p ≤ 0 .0001; d = 3.93, CI 95%: 2 .93 to 4.81 and d = 4.41, CI 95%: 3 .32 to 5. 35, respectively). In contrast, there was an increased somatic anxiety score post-game (16.12 ± 1 .19) compared to BL (9.79 ± 1 .10) and pre-game (10.54 ± 1.58) in the DG (p ≤ 0 .0001; d = 5.52, CI 95%: 4.24 to 6.64 and d = 3 .99, CI 95%: 2.98 to 4.88, respectively).

For cognitive anxiety, no differences were found between groups at BL (p = 0.999) and pre-game (p = 0.999) times. Additionally, no intra-group differences were found between BL and pre-game times in the VG (p = 0.492) and DG (p = 0.815). CSAI-2R in the post-game time was lower in VG than in DG (p ≤ 0.0001; d = 8.42, CI 95%: 6.57 to 10.00, Fig. **2C**). The mixed analysis of variance revealed a significant group-by-time interaction (F (2, 144) = 237.6; p ≤ 0.0001), main effects for group (F(1, 144) = 265.4; p ≤ 0.0001), and time (F(2, 144) = 53.62; p ≤ 0.0001) for anxiety state. The interaction analysis showed a reduction in cognitive anxiety levels post-game (6.70 ± 1.19) compared to BL (9.16 ± 1.12) and pre-game (9.95 ± 1.78) in the VG (p ≤ 0.0001; d = 2.13, CI 95%: 1.41 to 2.79 and d = 2.15, CI 95%: 1.42 to 2.80, respectively). Conversely, there was an increased cognitive anxiety score post-game (17.37 ± 1.34) compared to BL (9.37 ± 1.05) and pre-game (10.04 ± 1.75) in the DG (p ≤ 0.0001; d = 6.65, CI 95%: 5.14 to 7.94 and d = 4.70, CI 95%: 3.57 to 5.69, respectively).

### 3.2 Heart Rate Variability (HRV)

**Fig. (3):**
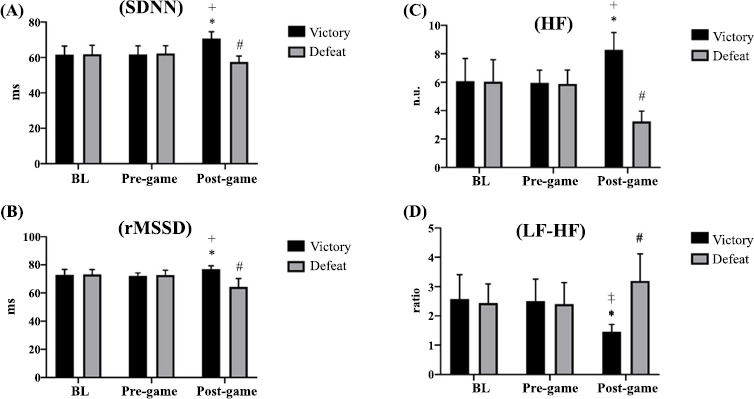
HRV in both groups. (**A**) SDNN: *Significant difference compared to BL and pre-game times (p ≤ 0.0001). **^+^**Significant difference compared to post-game time (p ≤ 0.0001), ^#^Significant difference compared to BL and pre-game times (p = 0.001 and p ≤ 0.0001, respectively); (**B**) rMSSD: *Significant difference compared to BL and pre-game times (p ≤ 0.0001; p = 0.003, respectively), **^+^**Significant difference compared to post-game time (p ≤ 0.0001), ^#^Significant difference compared to BL and pre-game times (p ≤ 0.0001); (**C**) HF: *Significant difference compared to BL and pre-game times (p ≤ 0.0001), **^+^**Significant difference compared to post-game time (p ≤ 0.0001), ^#^Significant difference compared to BL and pre-game times (p ≤ 0.0001); (**D**) LF-HF: *Significant difference compared to BL and pre-game times (p ≤ 0.0001), **^+^**Significant difference compared to post-game time (p ≤ 0.0001), ^#^Significant difference compared to BL and pre-game times (p = 0.004 and p = 0.002, respectively).

In the time domain, at baseline and pre-game, there were no differences in SDNN (standard deviation of normal-to-normal intervals) between the groups. However, SDNN was higher in the VG than the DG after the game (*p* ≤ 0.001; d = 3.73, CI 95%: 2.76 to 4.58). There was a significant group-by-time interaction (*F* (2, 144) = 39,82; *p* ≤ 0.001), with SDNN increasing in the VG (*p* ≤ 0.001; d = 2.13, CI 95%: 1.41 to 2.79 and d = 2.06, CI 95%: 1.35 to 2.71, respectively) but decreasing in the DG (*p* = 0.001; d = 1.05, CI 95%: 0.44 to 1.62 and p ≤ 0.001; d = 1.22, CI 95%: 0.61 to 1.81, respectively) from baseline and pre-game to post-game (Fig. **3A**).

Similar patterns were observed for rMSSD, which was higher in VG than in DG (*p* ≤ 0.001; *d* = 2.79, CI 95%: 1.97 to 3.52). There was a significant group-by-time interaction (*F* (2, 144) = 50,92; p ≤ 0.001), with rMSSD increasing in the VG (*p* ≤ 0.001; d = 1.27, CI 95%: 0.65 to 1.86 and *p* = 0.003; d = 2.15, CI 95%: 1.43 to 2.81, respectively) but decreasing in the DG (p ≤ 0.001; d = 1.82, CI 95%: 1.14 to 2.45 and d = 1.74, CI 95%: 1.06 to 2.36, respectively) from baseline and pre-game to post-game (Fig. **3B**).

Considering frequency domain measures, at baseline and pre-game, there were no differences in HF (high frequency) power between the groups. HF power was higher in the VG than the DG after the game (*p* ≤ 0.0001; d = 5.09, CI 95%: 3.89 to 6.14). There was a significant group-by-time interaction (*F* (2, 144) = 72,24; p ≤ 0.001), with HF increasing in the VG (*p* ≤ 0.001; d = 1.57, CI 95%: 0.92 to 2.18 and d = 2.20, CI 95%: 1.47 to 2.87, respectively) but decreasing in the DG (*p* ≤ 0.001; d = 2.33, CI 95%: 1.58 to 3.01 and d = 3.09, CI 95%: 2.23 to 3.86, respectively) from baseline/pre-game to post-game (Fig. **3C**). For LF/HF (low frequency/high frequency) ratio, there were no differences between groups at baseline and pre-game, but it was lower in the VG than the DG after the game (*p* ≤ 0.001; d = 2.59, CI 95%: 1.80 to 3.30). There was a significant group-by-time interaction (*F* (2, 144) = 27,55; *p* ≤ 0.001), with decreasing in LF-HF in the VG (*p* ≤ 0.001; d = 1.93, CI 95%: 1.23 to 2.57 and d = 1.98, CI 95%: 1.28 to 2.62, respectively), but increasing in the DG (*p* = 0.004; d = 0.95, CI 95%: 0.35 to 1.52 and *p* = 0.002; d = 1.03, CI 95%: 0.43 to 1.61, respectively) from baseline/pre-game to post-game (Fig. **3D**).

The study did not find any significant correlations between the HRV measures and the CSAI-2R (competitive state anxiety inventory) scores.

## 4 DISCUSSION

This current study aimed to investigate competitive anxiety and HRV responses before and after successful and unsuccessful matches (i.e., victory and defeat) in professional esports players. According to our hypothesis, winners had lower cognitive and somatic anxiety, higher self-confidence scores, and better HRV responses in comparison to losers who had the opposite results after the match.

Several studies have discussed the importance of monitoring anxiety and HRV in athletes. However, most studies focused on the impact of results in pre-competitive anxiety [23-26, 28, 49], HRV [27], or competitive anxiety responses before and after a competition [28], but not on the effects of results on competitive anxiety and HRV responses jointly. In line with that, our study revealed that victory and defeat interfere with HRV and anxiety responses.

Regarding anxiety responses, our findings indicated that VG experienced significantly reduced cognitive and somatic anxiety scores, along with increased self-confidence scores at the post-game time compared to baseline (BL) and pre-game times. In contrast, DG showed the opposite pattern, with significantly increased cognitive and somatic anxiety scores, as well as significantly reduced self-confidence scores at the post-game time compared to BL and pre-game times. Furthermore, there were no differences between BL and pre-game times, as well as between BL and post-game in both VG and DG on the Competitive State Anxiety Inventory 2 Revised (CSAI-2R).

Concerning heart rate variability (HRV), our results revealed a substantial increase in RR, SDNN, rMSSD, pNN50, and HF and a substantial decrease in LF and LF/HF in the VG. DG demonstrated a substantial decrease in RR, SDNN, rMSSD, and HF, and a substantial increase in LF and LF/HF. Moreover, there were no differences in BL and pre-game times, as well as between BL and post-game times in both VG and DG in any HRV measure. Our results align with the existing literature on HRV and competitive anxiety, emphasizing that different magnitudes of response may occur depending on the situation because of the robust correlation between the autonomic nervous system (ANS) and anxiety.

The results of a competition are considered a significant factor contributing to anxiety responses and HRV changes. Additionally, recent studies have underscored the importance of using HRV to monitor players, and the assessment of anxiety levels in high-performance sports is deemed crucial for emotional control throughout the competition. To the best of our knowledge, this study is the first to investigate the impact of victory and defeat on heart rate variability (HRV) responses and competitive anxiety. Despite this, it is reasonable to suggest that victory and defeat have positive and negative effects on HRV and anxiety behaviors. In this context, our results regarding anxiety levels are in line with a previous study by Fuentes-Garcia *et al.* [28], even though they specifically focused on competitive anxiety. The authors explored the influence of victory and defeat on pre-competitive anxiety in athletes during international tennis competitions. Significant differences were noted in cognitive anxiety and self-assurance levels when analyzing pre-game and post-game results among winners and defeated players. Winners encountered a notable decrease in cognitive anxiety and a rise in self-confidence following the match.

These findings reinforce our results, suggesting that esports players who emerge victorious tend to exhibit more adaptive responses in cognitive and somatic anxiety, self-confidence, and greater parasympathetic activation compared to losers. These differences can extend to HRV. In a similar vein, a study by Pena [27] demonstrated that junior tennis players who were defeated experienced a significant reduction in PNN50 after the match compared to the winners. These findings suggest that losers had higher sympathetic activity than winners after the match. Overall, anxiety can be regarded as a failure in the ability to inhibit cognitive, affective, behavioral, and physiological responses, leading to reduced vagal outflow and lowered HRV.

The neurovisceral integration model emphasizes the involvement of the prefrontal cortex in inhibitory processes mediated by the vagus nerve, which can be observed through measures of heart rate variability (HRV) [16]. An imbalance between sympathetic and parasympathetic activity, which is linked to task performance [50, 51], can give rise to significant alterations in prefrontal cortex functioning (responsible for executive functions) at various levels [52]. Consequently, these changes can impact an individual's psychological state, including the experience of anxiety [53].

There is a correlation between cognitive functioning, anxiety levels, and heart rate variability (HRV), as the connection between the brain and heart is constant and crucial for efficient and regulated functioning [54]. However, anxiety levels impact HRV through the vagus nerve (parasympathetic), which influences the sinoatrial node of the heart, resulting in an imbalance between sympathetic and parasympathetic activity [53, 55]. Moreover, given that players are consistently active, whether during training or competition, their performance can influence neurocardiac dynamics, and the outcome of a match (be it a win or a loss) can impact the establishment of a sympathetic/parasympathetic balance [50]. In esports, sustained demands on concentration, attention, and decision-making are required throughout the match to ensure task efficiency [8], and this demand leads to changes in anxiety levels and HRV, influencing positively or negatively on match performance. Furthermore, the concept of the polyvagal theory [56] emphasizes the significance of the myelinated vagus nerve in facilitating social interaction and communication, as well as inducing states of relaxation by suppressing sympathetic activity in the heart and modulating the hypo-pituitary-adrenal axis. When an individual perceives their environment as safe, there is an increase in vagal outflow, which promotes restoration, maintenance of internal balance, and engagement in social interactions. However, impairments in these neural processes can lead to possible damage to the perception that certain environments are safe, as well as difficulties in communication and social interaction, which can impair the performance of players during training and matches. Therefore, the link between anxiety and deficits in the inhibition of sympathetic function, as well as the difficulty in communication and social interaction, can be explained by the polyvagal theory.

Our findings have important implications for establishing the relationship between the reduction in HRV parameters with anxiety in professional esports players. Based on clinical studies, individuals with anxiety disorders have a higher attentional bias related to threatening cues when compared to individuals without anxiety disorders [57]. This pattern of behavior results in consistently elevated levels of corticotropin (released in response to stress) and cortisol, leading to a persistent suppression of parasympathetic activity (i.e., low vagal tone) [58]. Moreover, ongoing concerns may disrupt the regulation of cardiac autonomic function in the presence of threatening stimuli [14]. It is plausible that a decline in vagal function (which plays a critical role in regulating the hypothalamic-pituitary-adrenal axis) [59] contributes to heightened reactivity to stressors.

The inability to disengage from threat detection intensifies sympathetic activation due to the enduring reduction of parasympathetic activity (and subsequent long-term decreases in heart rate variability). Machado *et al.* [7] explored for the first time the impact of victory and defeat in playoff matches on competitive anxiety and HRV in professional esports players. Hence, more studies are required to better comprehend HRV patterns and emotional states (beyond stress and anxiety) in professional esports players, both prior to and following different game outcomes, such as victories, defeats, and potential draws. These studies hold significance in comprehending the dynamics of these factors over the course of a competitive season, thereby offering valuable insights for potential interventions aimed at facilitating the players' recovery between matches and throughout the entire competition.

The present study uncovered noteworthy, albeit preliminary, discoveries that hold relevance for professional esports players and coaches. Our results indicated that victory and defeat in playoffs provoke different responses in HRV and anxiety state. As a practical implication, we can use HRV as a monitoring tool for athletes during competition, especially in playoffs. The use of HRV in quantifying training loads is already established [31], as well as the analysis and organization of training programs [34]. This enables the evaluation, monitoring, recovery, and improvement of performance [60]. However, HRV was found to respond in accordance with the level of competitive anxiety influenced by environmental factors, such as victory or defeat. Thus, HRV plays an important role in this type of monitoring, as it is influenced by the emotional state [59].

In line with this, rMSSD is the most used HRV indicator at rest [61], as it is effective in identifying the parasympathetic level of the heart. Therefore, esports athletes and coaches should consider these results, as they can be utilized to evaluate, track, restore, and uphold effectiveness in training and game execution. This study has certain limitations. The examination of competitive anxiety and heart rate variability (HRV) in esports players during the game was not carried out, just before and after the games. Intra-match HRV and anxiety can provide insights into the athlete's psychophysiological state during the match. Without this data, the study may overlook critical fluctuations in stress and recovery that occur in real time, potentially skewing the relationship between HRV and performance outcomes. Moreover, HRV and anxiety are often used as a marker of fatigue and recovery. It is difficult to assess how fatigue accumulates throughout the match and how it correlates with performance metrics, leading to an incomplete picture of an athlete's condition. Also, there was no comparison of competitive anxiety and HRV parameters among players with different functions in the team. Furthermore, there was no assessment of a protective factor through psychotherapy or counseling that could prevent or alleviate symptoms early on.

For future studies, we recommend investigating the effects of ergogenic aids to enhance both neurocognitive and neuromotor performance, utilizing substances, such as caffeine [62] and non-invasive brain stimulation techniques like transcranial direct current stimulation (tDCS) [8, 63]. Furthermore, it would be beneficial to consider future studies that include measures during the matches and analyze differences according to team roles.

## CONCLUSION

This study aimed to analyze the behavior of competitive anxiety and HRV in different teams during playoff matches in professional esports players. Our results revealed that VG exhibited better HRV responses, indicating greater parasympathetic activation. VG also showed lower levels of cognitive and somatic anxiety and higher levels of self-confidence in the post-game time. In contrast, DG demonstrated worse HRV responses, indicating greater sympathetic activation, along with higher levels of cognitive and somatic anxiety and lower levels of self-confidence in the same post-game period. Future research should delve into analyzing HRV behavior and other emotional variables before, during, and after the conclusion of competitive esports matches with different outcomes (victory, defeat, and perhaps draw). Additionally, exploring the impact of various ergogenic aids on neurocognitive and neuromotor performance would be valuable.

## Data Availability

The data sets used and/or analysed during this study are available from the corresponding author [S.M.N] upon request.

## LIST OF ABBREVIATIONS

HRV: = Heart Rate Variability
VG: = Victory Group
DG: = Defeated Group
CSAI-2R: = Revised Competitive State Anxiety Inventory 2
